# Renoprotective effect of Tanshinone IIA against kidney injury induced by ischemia-reperfusion in obese rats

**DOI:** 10.18632/aging.204304

**Published:** 2022-10-20

**Authors:** He Tai, Xiao-Zheng Cui, Jia He, Zhi-Ming Lan, Shun-Min Li, Ling-Bing Li, Si-Cheng Yao, Xiao-Lin Jiang, Xian-Sheng Meng, Jin-Song Kuang

**Affiliations:** 1 School of Pharmacy, Liaoning University of Traditional Chinese Medicine, Dalian, China; 2 Department of Internal Medicine, Liaoning Provincial Corps Hospital of Chinese People’s Armed Police Forces, Shenyang, China; 3 Cardiovascular Surgery, Beijing Tsinghua Changgung Hospital, School of Clinical Medicine, Tsinghua University, Beijing, China; 4 Post-Doc Mobile Station, Liaoning University of Traditional Chinese Medicine, Shenyang, China; 5 Nephrology Laboratory, The Fourth of Affiliated Hospital of Guangzhou University of Traditional Chinese Medicine (Shenzhen Traditional Chinese Medicine Hospital), Guangzhou University of Traditional Chinese Medicine, Shenzhen, China; 6 Department of Graduate School, China PLA General Hospital, Beijing, China; 7 Key Laboratory of Ministry of Education for Traditional Chinese Medicine Viscera-State Theory and Applications, Liaoning University of Traditional Chinese Medicine, Shenyang, China; 8 Department of Endocrinology and Metabolism, The Fourth People’s Hospital of Shenyang, Shenyang, China

**Keywords:** renal ischemia-reperfusion, acute kidney injury, Tanshinone IIA, mitochondrial dysfunction, apoptosis

## Abstract

Objective: Obesity enhances the frequency and severity of acute kidney injury (AKI) induced by renal ischemia-reperfusion (IR). Tanshinone IIA (TIIA) pre-treatment was used to alleviate renal injury induced by renal IR, and whether TIIA can attenuate renal cell apoptosis via modulating mitochondrial function through PI3K/Akt/Bad pathway in obese rats was examined.

Methods: Male rates were fed a high-fat diet for 8 weeks to generate obesity, followed by 30 min of kidney ischemia and 24 h reperfusion induced AKI. The male obese rates were given TIIA (5 mg/kg.d, 10 mg/kg.d, and 20 mg/kg.d) for 2 weeks before renal IR.

Results: TIIA alleviated the pathohistological injury and apoptosis induced by IR. In addition, TIIA improved renal function, inflammatory factor, and balance of oxidation and antioxidation in obese rats after renal IR. At the same time, TIIA can inhibit cell apoptosis by improving mitochondrial function through the PI3K/Akt/Bad pathway. Mitochondrial dysfunction was supported by decreasing intracellular ATP, respiration controlling rate (RCR), mitochondrial membrane potential (MMP), and mitochondrial respiratory chain complex enzymes, and by increasing ROS, the opening of mitochondrial permeability transition pore (mPTP), and the mtDNA damage. The injury to mitochondrial dynamic function was assessed by decreasing Drp1, and increasing Mfn1/2; and the injury of mitochondrial biogenesis was assessed by decreasing PGC-1, Nrf1, and TFam.

Conclusions: Renal mitochondrial dysfunction occurs along with renal IR and can induce renal cell apoptosis. Obesity can aggravate apoptosis. TIIA can attenuate renal cell apoptosis via modulating mitochondrial function through PI3K/Akt/Bad pathway in obese rats.

## INTRODUCTION

As a common complication of the critically ill, acute kidney injury (AKI) presents a critical condition that can even cause danger to life [1]. As a significant health problem, AKI can be induced by the damage of ischemia/reperfusion (IR) induced by transplanted and native kidneys, and by increasing mortality or morbidity [2]. Because hyperuricemia, diabetes and hyperlipidaemia are tightly related with obesity which leads to insulin resistance (IR) and hypertension, they are thought to be chronic hyperinflammatory [3], which raises the incidence and severity of the diseases [4].

As a proverbial hazard factor for cardiovascular morbidity [5], hyperlipidemia can promote the process of all kinds of glomerular diseases [6]. Several lipid lowering tests have demonstrated that hyperlipidemia treatment may be an important means for managing chronic kidney diseases in early period [7]. In addition, the early period of AKI can occasionally combine with hyperlipidemia [8], while the pathobiological sense of hyperlipidemia in renal IR is indecipherable. Numerous studies demonstrated that the mechanism of IR (especially in the nerve and heart) is intently related to mitochondrial dysfunction [9, 10]. Nevertheless, there is no study available to explore the mitochondrial dysfunction of renal cells caused by renal IR.

As a main active composition of *Salvia miltiorrhiza* Bunge, the reduction of oxidant stress and inflammatory response are the major biological functions of Tanshinone IIA (TIIA, C_19_H_18_O_3_, CAS: 568-72-9) [11]. Besides, TIIA can play a protective function on myocardial ischemia [12]. Some researchers demonstrated that TIIA can repress the mitochondrial permeability transition pore (mPTP) opening, thus achieving the goal of liver protection and cardioprotection [13, 14]. TIIA pre-treatment attenuates IR-induced renal injury through antioxidizing capability and anti-inflammatory activity [15].

The survival effect is performed by a phosphoinositide-3 kinase (PI3K), which relies on Akt activation and kinase phosphorylation. Bad phosphorylation can be restrained. Besides, PI3K plays a significant role on the signaling of growth factors. With the activation of multiple physiochemical and cytokines factors, PI3K can generate myoinositol which can be seen as the second messenger; moreover, Akt performs a significant action on a lot of biological courses including cell growth, cell cycle, apoptosis, and metabolism [16]. Inhibiting mitochondria-mediated apoptosis can be achieved via activating PI3K/Akt/Bad signaling pathway [17]. Nevertheless, there are few studies about the renal protection via controlling mitochondrial function through the PI3K/Akt/Bad pathway.

In this context, the current research focuses on exploring a way of AKI (caused by IR) induced mitochondrial dysfunction in the kidney, employing PI3K/Akt/Bad, to cross the bridge between mitochondrial dysfunction and apoptosis to find a novel treatment plan.

## MATERIALS AND METHODS

### Chemicals and reagents

We dissolved TIIA (The first Pharmaceutical company in Shanghai) with deionized water to get the 5 mg/ml storage solution. All the storage solution must be used promptly.

### Experimental animals

SD male rats (weighing 180 g-220 g and aged 8 weeks) were applied to proceed our studies. The cages with a stable environment of 20 ± 3° C temperature, 45-65 % humidity, and dark (12 h) /light (12 h) (lights on 06:00 h-18:00 h) cycle were used to keep the rats. The rats were raised with water combined with pellet diet.

We divided 60 rats into 6 groups as following: sham operation group, IR/IR (obese) groups, and TIIA (5, 10, and 20 mg/kg.d) groups. Each group contained 10 rats. All the 60 rats in the 6 groups were given conventional maintenance feed for 2 weeks. Two groups of rats (IR and sham groups) were still fed with conventional feed for 8 weeks; however, the remaining 4 groups were given high-fat diet (HFD) feed for 8 weeks. The rats (sham group and IR/IR (obese) groups) were provided with deionized water. We supplied the rats in the remaining 3 groups with different dosages of TIIA for 2 weeks before renal IR. The compositions of HFD are as following: 13 % fiber, 11 % unsaturated fat, 25 % fat containing 44 % carbohydrate, 18 % protein, ash, and other constituents [18]. The rats which had 30 % increase in body weight were chosen for the subsequent research [19].

### Surgical process

We anesthetized rats (isoflurane through inhalation anesthesia), applied pinching paw/tail to assess the effect of anesthetic, and then dissected the abdomen in order to reveal the right kidney. Then, we detached renal pedicle to reveal vessels and ligating renal vessel with 3-0 silk suture, and then right kidney was resected. We exposed the left kidney and separated the vessels, followed by clamping off the renal artery (left) for 30 min in order to establish an ischemia model. After ischemia for 30 min, we removed clip to reperfuse 24 h. We observed the left kidneys for 15 min in order to ensure the reperfusion that showed the color resumed red [20]. We used 3-0 silk suture to close an abdominal incision and used the heat pad to preserve stable 37° C throughout the whole surgical process. Rats of sham group underwent the whole surgical procedure without clamping off the left artery [21].

### Serum renal function indexes, inflammatory factor analysis, renal activity of superoxide dismutase (SOD), and malondialdehyde (MDA) level

We used arterial blood to detect the renal function containing BUN and serum Cr with kits (Tiangen Biotech Co., Ltd., Beijing, China). Arterial blood (0.5 ml) was extracted from abdominal aorta (after the reperfusion period) to detect IL-1β and TNF-α via related kits (KHB, Shanghai, China). The concentration of MDA and the activity of SOD were detected using commercial kits (Beyotime, Shanghai, China).

### Histological assessment of the kidney using HE staining

The method of hematoxylin-eosin (HE) staining was managed according to previous experiments; after embedding with paraffin, the renal tissues were incised into thin sections (5 μm). The renal tissues were infiltrated with 4 % paraformaldehyde (24 h) and then diverted to 70 % ethanol. Then, H&E staining was applied to stain the renal tissues for detecting the renal tissues with light microscopy [21]. After immersing with 4 % paraformaldehyde (24 h) and then distracted to 70 % ethanol, the renal tissues were stained with H&E staining to observe renal tissues with light microscopy [21]. We assessed the degree of injury of kidney tissues (outer medulla, cortex, and inner medulla) by light microscopy in the means as follows: the grading of tissues injury was operated in terms of cell necrosis, cast formation in tubules, Bowman space enlargement, and vascular congestion. The highest grading of enlargement in Bowman space was compared with the sham operation group which was graded as 100% injury; the other rats were compared correspondingly. Vascular congestion, cell necrosis and tubular cast formation were graded in light of the percentage of the involved area as follows: 0, no damage; 1, 25 %; 2, 25–50 %; 3, 50 –75 %; and 4, 75 % [22].

### Observing mitochondria with transmission electron microscope

We took out the renal tissues from abdominal cavity immediately following anesthesia, and then sliced the renal tissues into snippets (1 mm^3^). The specific process was conducted based on previous studies [23].

### Apoptosis protein assessment of the renal tissues with immunofluorescence staining

Renal tissues were embedded with paraffin and then cut into thin sections (5 mm). Then, PBS was used to incubate the sections for 2 h at room temperature followed by incubation overnight (4° C) via the following two antibodies: rabbit anti-Bax (1: 50) and rabbit anti-Bcl-2 (1: 100). Finally, we used the secondary antibodies (Servicebio, Wuhan, China) to incubate the thin sections for 2 h at 4° C, and used a fluorescence microscope (Leica) to observe the results [23].

### TUNEL staining

We used the TUNEL assay to examine apoptosis. As recorded in HE staining [24], thin sections (5 μm) were used for such staining. After deparaffinization and rehydration, the samples were added to TUNEL reaction mixture using the protease K (10 μg/ mL) for handling the sections (15 min), and then were incubated at 37° C for 60 min in dark. Following cleaning, DAPI (0.1 μg/mL) was employed to stain cell nuclei. Then, a fluorescence microscope (T2130, Solarbio, Beijing, China) was employed to observe the samples. We observed eight random visual fields in a blinded manner to calculate the number of TUNEL-positive cells.

### Caspase-3/9 activity

A fluorescent caspase-specific Detection Kit (Solarbio, Beijing, China) was employed to examine the activity of caspase-3/9 in the renal tissues. We added 10 mg portion of renal tissue proteins to the reaction buffer for incubation (2 h) at 37° C. Then, a fluorimeter (405 nm) was employed to quantify the releasing of Enzyme-catalyzed.

### Preparation of mitochondria suspension

According to previous studies on the liver [25], the rats were sacrificed after anesthetizing with 120 mg/kg thiopental sodium through intra-peritoneal injection and drawing blood from the abdominal aorta. Then, the kidney was quickly removed and laid on the ice-cold isolated buffer (PH 7.4). After shearing, renal tissues (50-100 mg) were rinsed in an isolated buffer. In order to preserve mitochondrial wholeness, the whole procedure was operated at 4° C. With centrifugation (700×g) for 10 min, we collected supernate then centrifuged (7000×g) for 10 min twice. Then, we threw away supernate, resuspending to wash the mitochondria pellet with isolated buffer (5 ml) and centrifuged (7000×g, 10 min) twice. We obtained clean mitochondrial suspension for preservation in mitochondrial preservation solution (1 mM EDTA, 20 mM sucrose, 5 mM HEPES, 100 mM KCl, 10 mM KH_2_PO_4_, and 2 mM MgCl_2_) to generate a mitochondria suspension (the protein concentration is 5 mg/ml) which was placed on ice for timely use. Bicinchoninic acid (BCA) test kit (Beyotime, Shanghai, China) was used to detect the concentration of protein in the renal mitochondrial suspension for ensuring a stable protein level (100-1000 μg/ml). We used mitochondria suspension to measure MMP [25], the opening of mPTP [26], damaged mtDNA, ROS [27], mitochondrial oxygen consumption rate [28], RCR [28], mitochondrial respiratory chain complex enzymes (I, II, III, IV, and V) [23], and ATP.

### RNA extraction, cDNA synthesis, and real-time RT-qPCR

We used a Trizol kit to separate total genome RNA. We used spectrophotometry (260 nm) to assess the quality of isolated RNA. M-MLV Reverse Transcriptase Kit and total RNA (1 μg) were used to operate the reverse transcription. In brief, total reaction volume (40 μL) was applied in PCR system according to the following reaction process: at 72° C for 3 min, 42° C for 90 min, and 70° C for 15 min, and then preservation at 4° C. The method of RT-qPCR was employed to measure the copy number of specific gene transcription level with templates of cDNA. The PCR was operated on a Rotor-Gene Q Sequence Detection System with SYBR Premix Ex TaqII [29] in 20 μL system (including 10 μL SYBR Premix Ex Taq II + 1μL synthetic cDNA and 0.5 μM primers) according to the following procedure: 95° C (10 min), 95° C (10 s), 40 cycles, 60° C (15 s), 72° C (20 s), and 72° C (10 min). The level was counted with GAPDH as control [30]. The applied PCR primer sequences (two pairs) were presented in Table 1.

**Table 1:** Sequence of primers for RT-PCR and long PCR.

**Target gene**	**Primer sequence**	**Size (bp)**	**Tm (° C)**
Mfn1	Forward: 5’-GGGAAGACCAAATCGACAGA-3’	152	57
	Reverse: 5’-CAAAACAGACAGGCGACAAA-3’		57
Mfn2	Forward: 5’-GAGAGGCGATTTGAGGAGTG-3’	165	58
	Reverse: 5’-CTCTTCCCGCATTTCAAGAC-3’		56
Drp1	Forward: 5’-GCCCGTGGATGATAAAAGTG-3’	215	56
	Reverse: 5’-TGGCGGTCAAGATGTCAATA-3’		56
PGC-1α	Forward: 5’-GGACGAATACCGCAGAGAGT-3’	201	59
	Reverse: 5’-CCATCATCCCGCAGATTTAC-3’		56
Nrf1	Forward: 5’-AAACCGAACACATGGCTACC-3’	168	58
	Reverse: 5’-CTGCCGTGGAGTTGAGTATG-3’		58
Tfam	Forward: 5’-TCACCTCAAGGGAAATTGAAG-3’	241	55
	Reverse: 5’-CCCAATCCCAATGACAACTC-3’		56
Long Fragment	Forward:5’-AAAATCCCCGCAAACAATGACCACCC-3’	13400	72
	Reverse: 5’-GGCAATTAAGAGTGGGATGGAGCCAA-3’		72
Shrot Fragment	Forward: 5’-CCTCCCATTCATTATCGCCGCCCTGC-3’	235	60
	Reverse: 5’-GTCTGGGTCTCCTAGTAGGTCTGGGAA-3’		60
Bax	Forward: 5’-GCGATGAACTGGACAACAAC-3’	200	57
	Reverse: 5’-GATCAGCTCGGGCACTTTAG-3’		58
Bcl-2	Forward: 5’-CGAGTGGGATACTGGAGATGA-3’	236	58
	Reverse: 5’- GACGGTAGCGACGAGAGAAG-3’		59
Caspase-3	Forward: 5’-CCCATCACAATCTCACGGTAT-3’	195	57
	Reverse: 5’-GGACGGAAACAGAACGAACA-3’		58
Caspase-9	Forward: 5’-GCCTCTGCTTTGTCATGGAG-3’	181	56
	Reverse: 5’-AGCATGAGGTTCTCCAGCTT-3’		56
PI3K	Forward: 5’-TCACCTCCCTGATTGGCTAC-3’	220	58
	Reverse: 5’-CCACGATGGATGACAATGAA-3’		55
Akt	Forward: 5’-CGAGTCCCCACTCAACAACT-3’	231	59
	Reverse: 5’-GGTGAACCTGACCGGAAGTC-3’		60
Bad	Forward: 5’-GAGCTGACGTACAGCGTTGA-3’	153	60
	Forward: 5’-CCTGAGGGCTGTCCAGTAAC-3’		60
PARP	Forward: 5’-AAGCCTGGCACTAAGTCGAA-3’	164	59
	Forward: 5’-ATAGAGTAGGCGGCCTGGAT-3’		60
Cyc-c	Forward: 5’-GGACAGCCCCGATTTAAGTA-3’	121	57
	Forward: 5’-TCAATAGGTTTGAGGCGACAC-3’		58
GAPDH	Forward: 5’- AGGTCGGTGTGAACGGATTTG -3’	20	58
	Reverse: 5’- GGGGTCGTTGATGGCAACA-3’		58

### Protein detection

We used RIPA Lysis Buffer to extract the total proteins from renal tissues. The protein level was detected using BCA Protein kit. An equal gauge of total protein was dominated by sodium dodecyl sulfate-polyacrylamide gel electrophoresis (8–12 %); and then the proteins were transferred to the PVDF membrane. After sealing in skim milk solution, the membrane was incubated overnight respectively with anti-GAPDH, anti-PI3K, anti-p-Akt, anti-Akt, anti-p-Bad, anti-Bad, anti-Bax, anti-Bcl-2, anti-Cyt-c, anti-PARP, anti-caspase-9/3, anti-Drp1, anti-Mfn1/2, anti-Tfam, anti-PGC-1, and anti-Nrf1 antibodies (Table 2 demonstrated all the antibodies), and then incubated with secondary HRP-conjugated goat anti-rabbit antibodies. We used an augmented chemiluminescence kit to visualize the proteins, and performed the densitometric analysis using Alpha View software.

**Table 2:** Antibodies used in the study.

**Antibodies**	**Manufacturer**	**Catalogue No.**	**Observed MW**	**Dilution**
Anti-PI3K	Proteintech	67071-1-1g	110 KDa	1:10,000
Anti-p-Akt	Proteintech	66444-1-1g	62 KDa	1:10,000
Anti-Akt	Proteintech	10176-2-AP	56 KDa	1:5,000
Anti-p-Bad	Cell Signaling Technology	5284S	23 KDa	1:1,000
Anti-Bad	Proteintech	10435-1-AP	18 KDa	1:2,500
Anti-Bcl-2	Proteintech	26593-1-AP	26 KDa	1:2,500
Anti-Bax	Proteintech	50599-2-1g	26 KDa	1:10,000
Anti-Caspase-3	Proteintech	19677-1-AP	32 KDa	1:2,000
Anti-cleaved-Caspase-3	Abcam	ab49822	17 KDa	1:500
Anti-Caspase-9	Proteintech	10380-1-AP	47 KDa	1: 1,000
Anti-cleaved-Caspase-9	Affinity Biosciences	AF5240	10 KDa	1: 1,000
Anti-PARP1	Proteintech	13371-1-AP	89 KDa	1:2,000
Anti-Cyt-c	Proteintech	12245-1-AP	13 KDa	1:2,000
Anti-Mfn1	Proteintech	13798-1-AP	86 KDa	1:1,000
Anti-Mfn2	Proteintech	12186-1-AP	86 KDa	1:5,000
Anti-Drp1	Proteintech	10656-1-AP	27 KDa	1:4,000
Anti-PGC1a	Proteintech	66369-1-1g	100 KDa	1:5,000
Anti-Nrf1	Proteintech	12482-1-AP	67 KDa	1:2,500
Anti-Tfam	Proteintech	22586-1-AP	25 KDa	1:5,000
Anti-GAPDH	Proteintech	60004-1-1g	36 KDa	1:10,000

### Statistical analysis

SPSS statistical package was employed to operate statistical analysis. Data was displayed in form of mean ± standard deviation. One-way analysis of variance was conducted to compare among 6 separate groups; while the two-to-two comparison among groups was employed to analyse variance. We used Lsd-t test to perform multiplicate comparison between 6 groups. The difference with *p* < 0.05 was defined to be statistically significant.

### Data availability statement

The datasets analyzed during the current study are available from all the authors on reasonable request.

## RESULTS

### TIIA improved renal function, inflammatory factor, SOD, and MDA after renal IR of obese rats

**Figure 1:**
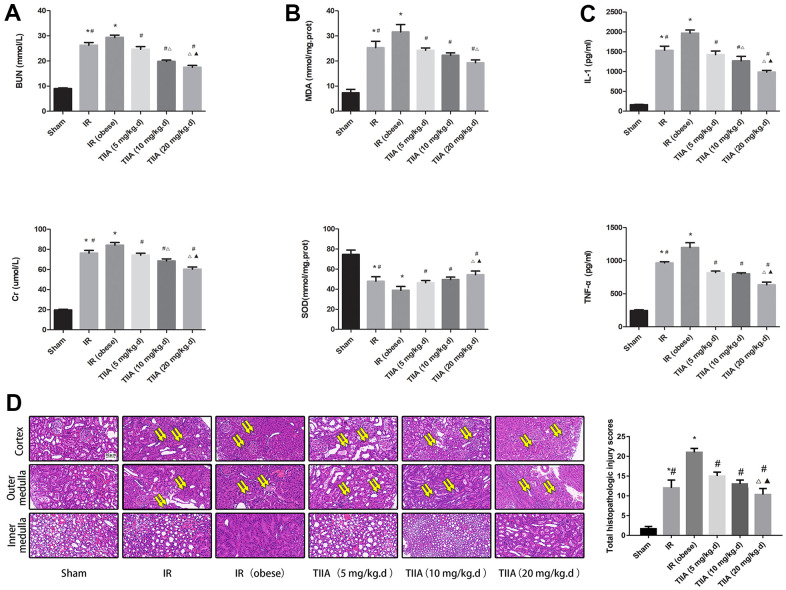
**Tanshinone IIA (TIIA) improved the renal function, malondialdehyde (MDA), superoxide dismutase (SOD), inflammatory factor, and renal architecture after renal ischemia-reperfusion (IR).** Rats were pre-treated with TIIA followed by removing the right kidney and clamping of the left renal artery for 30 min and reperfusion for 24 h. Sham rats were used as control. Renal function (**A**), MDA, SOD (**B**), inflammatory factor (**C**), and renal architecture (**D**) were evaluated under different groups. Representative photomicrographs of renal histology (**D**), the scale bars represent a length of 50 μm on histology, swollen renal tubules (paired yellow arrow). Data are shown as mean ± SD. *p < 0.05 versus sham group, ^#^*p* < 0.05 versus IR (obese) group, ^∆^*p* < 0.05 versus TIIA (5 mg/kg.d) group, **^▲^***p* < 0.05 versus TIIA (10 mg/kg.d) group. (n=3).

In order to further evaluate the renal damage and protective action of TIIA, we measured the renal function (Figure 1A), oxidizing substance (SOD and MDA) (Figure 1B), and inflammatory factor (Figure 1C).

BUN and Cr were elevated in IR/IR (obese) groups (*p* < 0.05), while BUN and Cr were reduced via pre-treatment with TIIA, especially with the dose of 20 mg/kg.d TIIA (*p* < 0.05) (Figure 1A). Rats renal SOD was decreased in IR/IR (obese) groups, especially in the IR (obese) group (*p* < 0.05). Nevertheless, it was elevated via pre-treatment with TIIA, especially with the dose of 20 mg/kg.d TIIA (*p* < 0.05) (Figure 1B). Inflammatory factor (TNF-α and IL-1β) levels of the rats were elevated in the IR/IR (obese) groups, especially in the IR (obese) group (*p* < 0.05), yet were decreased via pre-treatment with TIIA, especially with the dose of 20 mg/kg.d TIIA (*p* < 0.05) (Figure 1C).

### TIIA improved pathological structure of kidney following renal IR in obese rats

We evaluated protective action of TIIA on renal damage following IR of obese rats and observed kidney tissue using the hematoxylin-eosin (HE) staining (Figure 1D). IR (obese) and IR led to a significant expansion in Bowman space. Cortical thick ascending limb of the loop of Henle and proximal tubule had a significant injury. IR (obese) and IR caused significant injury in the outer medulla (tubular cast formation and vascular congestion, medullary thick ascending limb of loop of Henle, and perirenal tubule) and inner medulla (vascular congestion and tubular cast formation). Pre-treatment with TIIA significantly decreased the degree of renal tissue injury in the inner, outer medulla, and cortex. IR (obese) and IR led to a significant increase in total histopathologic scale (*p* < 0.05). The total histopathologic scale was reduced via pre-treatment with TIIA, especially with the dose of 20 mg/kg.d TIIA (*p* < 0.05), where more renal tubules were swollen (paired yellow arrow) were showed following IR (Figure 1D) and Table 3.

**Table 3:** Summary of the degree of kidney histopathologic damages in the six groups rats.

**Histopathologic damages**	**Sham**	**IR**	**IR (obese)**	**TIIA** **(5 mg/kg.d)**	**TIIA** **(10 mg/kg.d)**	**TIIA** **(20 mg/kg.d)**
Cortex						
bowman capsule	0.33±0.58	3.00±1.00	4.00±0.00	3.67±0.58	3.00±0.00	2.67±1.54
proximal tubal	0.33±0.58	1.33±0.58	2.67±0.58	1.67±0.58	1.67±0.58	1.33±0.58
thick ascending limb of Henle’s loop	0.00±0.00	1.00±0.00	2.33±0.58	1.33±0.58	1.00±0.00	1.00±0.00
Outer medulla						
thick ascending limb of Henle’s loop	0.33±0.58	1.33±0.58	3.33±0.58	2.33±0.58	1.67±0.58	1.33±0.58
vascular congestion	0.00±0.00	1.00±0.00	2.00±1.00	1.33±0.58	1.33±0.58	1.00±0.00
tubular protein cast	0.00±0.00	2.00±1.00	1.67±1.15	1.33±0.58	1.00±0.00	1.00±0.00
Inner medulla						
vascular congestion	0.33±0.58	1.00±0.00	2.33±0.58	1.67±0.58	1.67±0.58	1.00±0.00
tubular protein cast	0.33±0.58	1.33±0.58	2.67±0.58	1.67±0.58	1.67±0.58	1.00±0.00
Total histopathologic injury scores	1.67±0.58	12.00±2.00*^#^	21.00±1.00*	15.00±1.00^#^	13.00±1.00^#^	10.33±1.53^#∆▲^

Note: *p < 0.05 versus sham group, ^#^*p* < 0.05 versus IR (obese) group, ^∆^*p* < 0.05 versus TIIA (5 mg/kg.d) group, ^▲^*p* < 0.05 versus TIIA (10 mg/kg.d) group. (n=3).

### TIIA reduces renal cell apoptosis led by renal IR in obese rats

**Figure 2:**
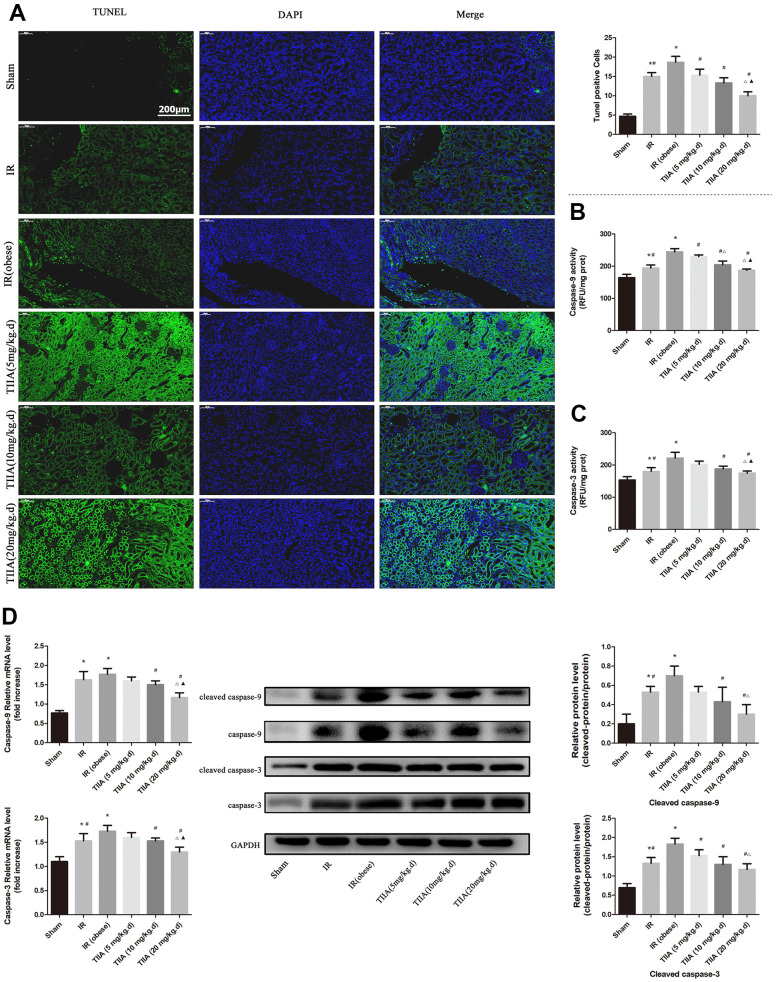
**Tanshinone IIA (TIIA) inhibited renal cells apoptosis after renal ischemia-reperfusion (IR).** Rats were pre-treated with TIIA followed by removing the right kidney and clamping of the left renal artery for 30 min and reperfusion for 24 h. Sham rats were used as control. Sham rats were used as control. Representative apoptosis of renal cells, TUNEL positive cells, the scale bars represent a length of 200 μm on histology (**A**), the activity of renal caspase-9/3 (**B**, **C**), and the protein expression of cleaved caspase-9/3 (**D**) were evaluated under different groups. Data are shown as mean ± SD. *p < 0.05 versus sham group, ^#^*p* < 0.05 versus IR (obese) group, ^∆^*p* < 0.05 versus TIIA (5 mg/kg.d) group, **^▲^***p* < 0.05 versus TIIA (10 mg/kg.d) group. (n=3).

**Figure 3:**
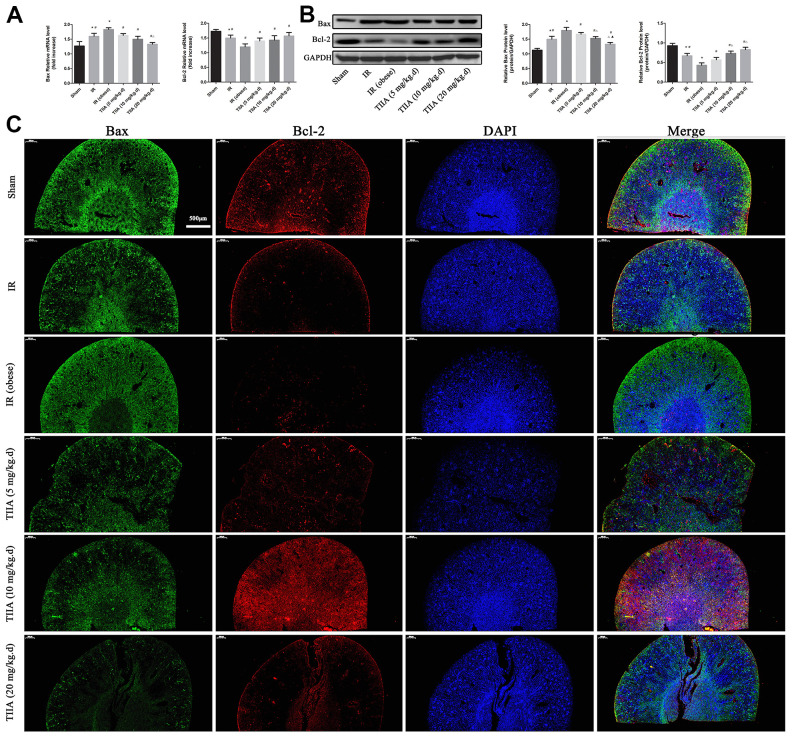
**Tanshinone IIA (TIIA) decreased Bax and increased Bcl-2.** The expression of Bax and Bcl-2 in mRNA (**A**) and protein (**B**) level. Immunofluorescence results of Bax (green) and Bcl-2 (red) expression in nephridial tissue, the scale bars represent a length of 500 μm on histology (**C**). *p < 0.05 versus sham group, ^#^*p* < 0.05 versus IR (obese) group, ^∆^*p* < 0.05 versus TIIA (5 mg/kg.d) group, **^▲^***p* < 0.05 versus TIIA (10 mg/kg.d) group. (n=3).

We used the TUNEL assay to evaluate the protective action of TIIA on renal tissue cell apoptosis induced by IR (Figure 2A). Apoptotic cell numbers of renal tissues in IR/IR (obese) groups were increased compared with those in sham group (*p* < 0.05). However, pre-treatment with TIIA reduced cell apoptosis in renal tissues (Figure 3A). Caspase-9/3 was significantly activated in the IR/IR (obese) groups compared with sham group (*p* < 0.05). Nevertheless, pre-treatment with TIIA decreased the activity of caspase-9/3 (Figure 2B, 2C). The cleaved caspase-9/3 in renal tissues in IR/IR (obese) groups were elevated compared with sham group (*p* < 0.05). Nevertheless, pre-treatment with TIIA reduced the relative levels of cleaved caspase-9/3 (*p* < 0.05) (Figure 2D).

### TIIA improved the expression of apoptosis-related genes led by renal IR of obese rats

Bax in mRNA and protein levels were increased in IR/IR (obese) groups (*p* < 0.05). Bax in mRNA and protein levels were decreased following pre-treatment with TIIA (*p* < 0.05). Bcl-2 in mRNA and protein levels were decreased in IR/IR (obese) groups (markedly reduced in the IR (obese) group) (*p* < 0.05) (Figures 3A, 3B). The immunofluorescence results of Bax/ Bcl-2 levels of renal tissues demonstrated that Bax levels increased. However, Bcl-2 levels were reduced in IR/IR (obese) groups. Bax level was reduced, yet Bcl-2 was increased, following the pre-treatment with TIIA (Figure 3C).

### TIIA improved mitochondrial function and mitochondrial morphology after renal IR of obese rats

**Figure 4:**
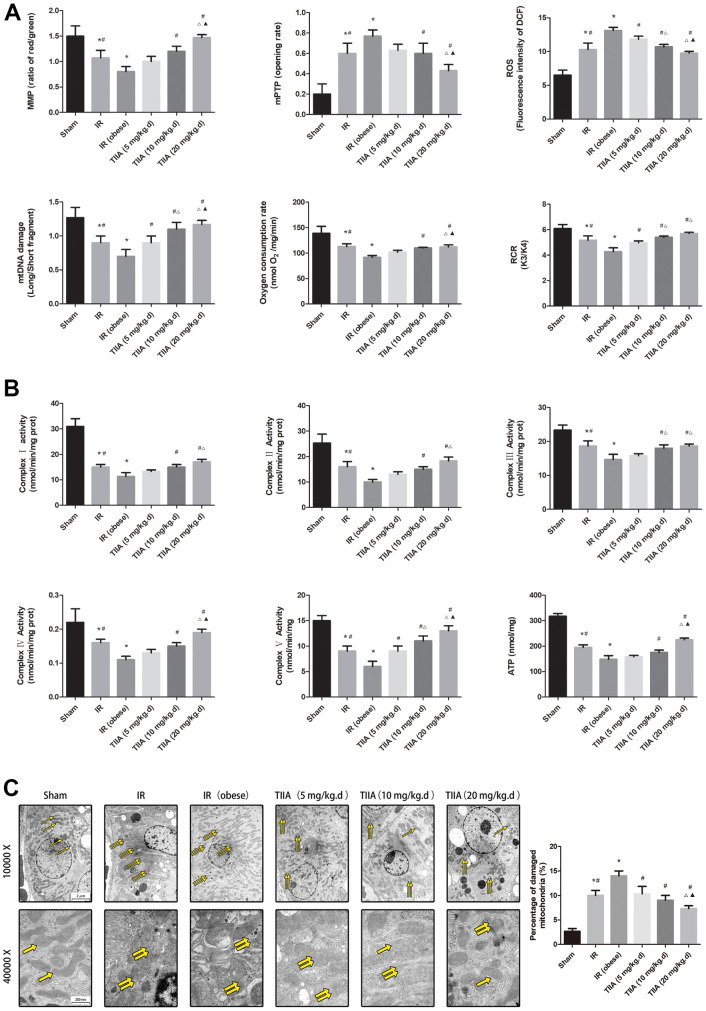
**Tanshinone IIA (TIIA) preserved renal mitochondrial function in renal ischemia-reperfusion (IR)-induced renal injury.** The MMP (ratio of red/green), the opening of mPTP (%), the mitochondrial ROS, the mtDNA damage (ratio of long/short fragments), the mitochondrial RCR, mitochondrial oxygen consumption rate (**A**), the mitochondrial respiratory chain complex enzymes (I, II, III, IV, and V), and ATP (**B**) were recorded above. Electron microscope pictures (10 000×, 40 000×) of renal tissue after IR, the scale bars represents a length of 2 μm and 200 nm on tissues respectively. Abnormal mitochondrial (paired yellow arrow) morphology show that mitochondrial membrane rupture or swellings, normal mitochondrial (single yellow arrow) morphology type show that mitochondrial membrane smooth and inner carinulae distinct (**C**), and percentage of damaged mitochondria (**C**). Data are shown as mean ± SD. *p < 0.05 versus sham group, ^#^*p* < 0.05 versus IR (obese) group, ^∆^*p* < 0.05 versus TIIA (5 mg/kg.d) group, ^F070^*p* < 0.05 versus TIIA (10 mg/kg.d) group. (n=3).

IR increased ROS concentration and opening of mPTP (%) compared with sham group (*p* < 0.05). The ROS concentration was decreased via pre-treatment with TIIA (*p* < 0.05). IR decreased RCR, mitochondrial oxygen consumption rate, and MMP compared with the sham group (*p* < 0.05). Pre-treatment with TIIA significantly increased these factors (*p* < 0.05). The ratio of long/short fragments was reduced in IR/IR (obese) groups (*p* < 0.05). Pre-treatment with TIIA increased the ratio of long/short mtDNA fragments (*p* < 0.05) (Figure 4A). IR reduced ATP and mitochondrial respiratory chain complex enzymes compared with sham group (*p* < 0.05). Pre-treatment with TIIA significantly elevated the activity of these enzymes (*p* < 0.05) (Figure 4B). We assessed mitochondrial function to further evaluate renal damage and protective action of TIIA. A transmission electron microscope was used to observe the changes in the morphology of the mitochondria (Figure 4C). The photographs of electron microscopic (10,000× and 40,000×) of renal tissues demonstrated that renal cells in IR/IR (obese) groups displayed abnormal mitochondrial morphology in the form of swelling and even membrane rupture (paired yellow arrows) following renal IR. The sham group displayed normal mitochondrial morphology (single yellow arrow). The percentage of damaged mitochondria of renal tissue in IR/IR (obese) groups was increased (*p* < 0.05). Nevertheless, the pre-treatment with TIIA can reduce the percentage of damaged mitochondria (Figure 4C).

### TIIA improved mitochondrial dynamics and biogenesis induced by renal IR in obese rats

**Figure 5:**
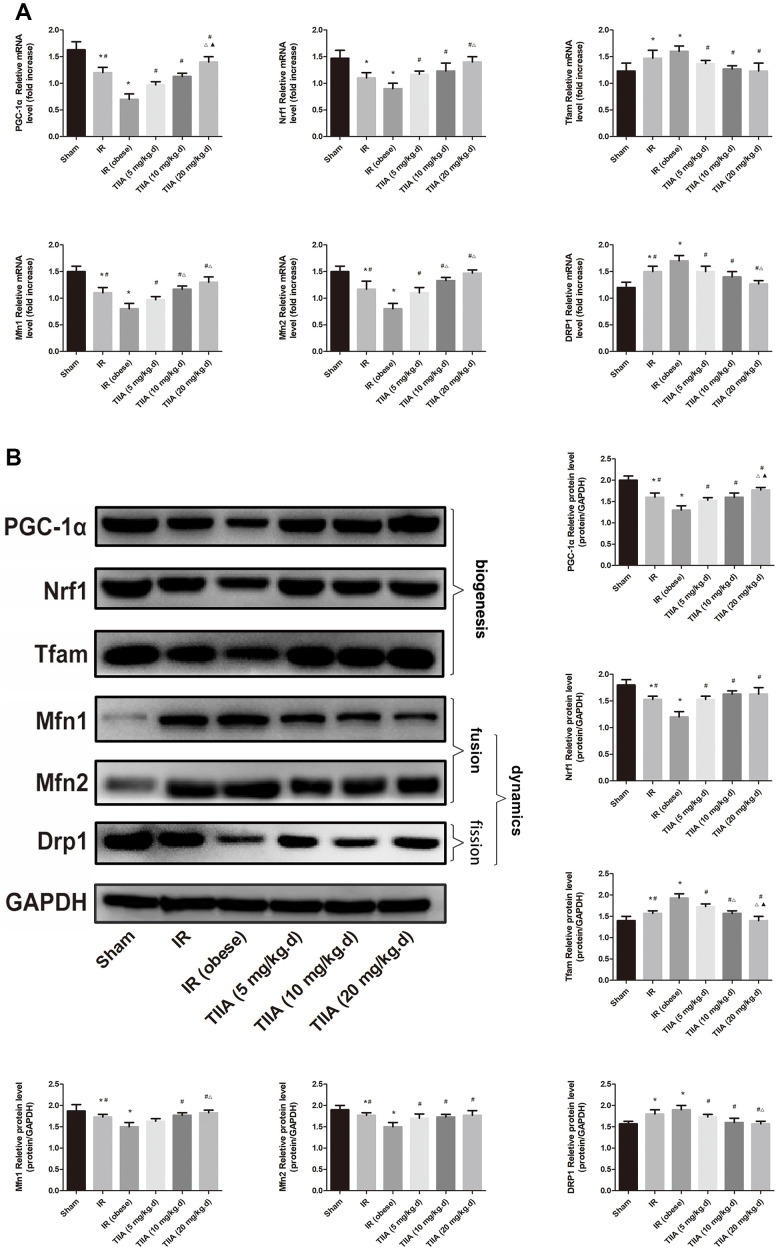
**Tanshinone IIA (TIIA) preserved mitochondrial biogenesis and dynamics in renal ischemia-reperfusion (IR)-induced renal injury.** The expression of PGC-1α, Nrf1, and Tfam in mRNA and protein level. The expression of Mfn1, Mfn2, and Drp1 in mRNA (**A**) and protein (**B**) levels. *p < 0.05 versus sham group, ^#^*p* < 0.05 versus IR (obese) group, ^∆^*p* < 0.05 versus TIIA (5 mg/kg.d) group, ^F070^*p* < 0.05 versus TIIA (10 mg/kg.d) group. (n=3).

The nucleo respiratory factor1 (Nrf1), PPARγ coactivator-1-α (PGC-1α), and transcription factor A of mitochondrial (Tfam) were chosen to indicate biogenesis. Mitofusins (Mfns (fusion); Mfn1/2) and dynamin-related protein 1 (Drp1; fission). The results demonstrated that Nrf1, PGC-1α, Drp1, and Tfam mRNA levels were reduced in the IR/IR (obese) groups (*p* < 0.05). Pre-treatment with TIIA increased these mRNA levels (*p* < 0.05). mRNA levels of Mfn1/2 were elevated in the IR/IR (obese) groups (*p* < 0.05). The mRNA levels of those indexes were reduced via pre-treatment with TIIA (*p* < 0.05) (Figure 5A). Western blot of dynamics and biogenesis indexes demonstrated similar results (Figure 5B).

### TIIA activated the PI3K/Akt/Bad pathway *in vivo*

**Figure 6:**
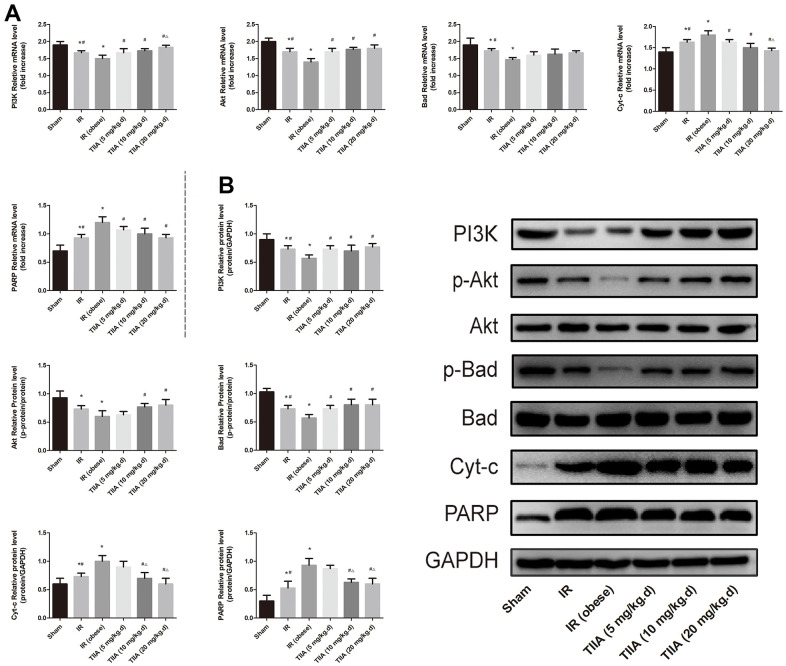
**Tanshinone IIA (TIIA) modulated PI3K/Akt/Bad pathway.** The expression of PI3K, p-Akt, Akt, p-Bad, Bad, Cyt-c, and PARP in mRNA (**A**) and protein (**B**) levels. *p < 0.05 versus sham group, ^#^*p* < 0.05 versus IR (obese) group, ^∆^*p* < 0.05 versus TIIA (5 mg/kg.d) group, ^F070^*p* < 0.05 versus TIIA (10 mg/kg.d) group. (n=3).

IR/ IR (obese) increased the mRNA level of PARP and Cyt-c significantly (*p* < 0.05). The mRNA level of these indexes was decreased following pre-treatment with TIIA (*p* < 0.05). IR/ IR (obese) decreased the mRNA level of PI3K, Bad, and Akt significantly (*p* < 0.05). The mRNA level of these indexes was increased following pre-treatment with TIIA (*p* < 0.05) (Figure 6A). Western blot of those genes showed similar results (Figure 6B). TIIA activated phosphorylation of Akt and Bad (increasing ratio of p-Akt/Akt and p-Bad/Bad) (Figure 6B).

## DISCUSSION

The new findings of our research include the aggravation of renal injury following I/R when accompanied with obesity, the potential protective action, and mechanisms of TIIA on renal I/R damage. We confirmed this using *in vivo* model. As compared with non-obesity rats, mitochondrial function was hypofunction in obese rats. This became more obvious following I/R. Cell injury and apoptosis were shown to be more severe in obese rats following I/R. According to the above results, it is the first study that explored the combined influence of obesity and renal I/R on mitochondrial function in renal tissues.

Oxidative stress raised the levels of ROS and inflammation via releasing proinflammatory mediators during the reperfusion period. These indexes perform a meaningful function on the pathophysiological process of renal IR. For injury mechanism of renal IR above, a few anti-inflammatory and anti-oxidants medicaments, which can increase the survivability of IR damage [31], were found to be effectively decreased.

Modern medical techniques have confirmed that as a well-known herbal medicine, *Salvia miltiorrhiza* Bunge can eliminate the toxic substance in the blood, accelerate the activity of a fibrinolytic enzymes, reduce the blood viscosity, and protect the cardiovascular system [32]. TIIA is the primary active component in *Salvia miltiorrhiza* Bunge. TIIA pre-treatment can relieve renal damage caused by IR via reducing the expression of inflammation, caspase-9/3, and myeloperoxidase (MPO) [15]. TIIA can decrease the release of Cyt-c and the production of ROS, inactivate caspase-3, reduce apoptotic, and inhibit mPTP opening. Inhibited opening of mPTP is the key for improving mitochondrial function. so it has been fundamental to hint that mPTP can be seen as an objective for curing, which can be ameliorated at the beginning of reperfusion [33]. It is common knowledge that mitochondrial dysfunction (especially the opening of mPTP) performs an important function in raising damage after renal IRI [34]. The opening of mPTP causes decreased MMP and the mitochondrial swelling which inhibits oxidative phosphorylation. The opening of mPTP is performed via binding to the CyP-D. Previous studies on the heart have shown that CsA repressed IRI via binding CyP, independently of anti-calcineurin properties, hence repressing mPTP opening [35]. In this research, we assumed TIIA can perform a protective role in AKI induced by IR via improving the mitochondrial function (inhibiting mPTP opening) and downregulation of inflammation.

Our research showed that feeding rats with HFD for 8 weeks induced obesity in modality of weight gain and perirenal fat cumulation. HFD could lead to inflammation, hyperlipidemia, and imbalance of oxidative stress but failed to injury renal histological structure and function [21]. However, with the condition of IR, hyperlipidemia could aggravate the damage of renal histological structure compared with non-hyperlipidemia in our study. In addition, we found that IR could induce the imbalance between SOD and MDA (reducing the SOD and increasing MDA); and with the condition of IR, hyperlipidemia could aggravate the imbalance. When using the TIIA as the protective agent, the imbalance induced by renal IR combined with hyperlipidemia could be improved (Figure 1B). HE staining for rat renal tissue showed that IR and IR (obese) led to significant enlargement in Bowman space. The proximal tubule and cortical thick ascending limb of the loop of Henle had a significant injury. IR and IR (obese) caused significant injury in the outer medulla (vascular congestion, tubular cast formation, perirenal tubule, and medullary thick ascending limb of loop of Henle) and inner medulla (tubular cast formation and vascular congestion). Pre-treatment with TIIA could significantly decrease the degree of damage (Figure 1D and Table 3). TUNEL assay demonstrated that renal IRI led to cell apoptosis (Figure 2A). Nevertheless, TIIA reduced the number of apoptotic cells (Figure 2A).

Caspase-9/3 are the influential performers of apoptosis correlated to terminal period of apoptosis. The activity of caspase-9/3, which was detected in the present study, is a significant signal of apoptosis. IR and IR+obesity can significantly activate Caspase-9/3, particularly the IR+obesity (*p* < 0.05). Nevertheless, TIIA decreased the activity of caspase-9/3 in renal tissues (Figure 2B, 2C). We detected the relative levels of cleaved caspase-9/3 (Figure 2D) as the active form of caspase-9/3. Cleaved caspase-9/3 can guide apoptosis, while IR can increase the levels of cleaved caspase-9/3. However, pre-treatment with TIIA can reduce the levels of cleaved caspase-9/3 (*p* < 0.05) (Figure 2D). Unlike other organs [36], kidney is surrounded by perirenal adipose tissue, which may also be the primary source for inducing a subclinical inflammatory response in patients with chronic kidney disease [37]. In our study, I/R increased the TNF-α and IL-1β values. With the condition of IR, hyperlipidemia increased the IL-1β and TNF-α levels further, and they were reduced by pre-treatment with TIIA (Figure 1C). As the leading cause of the AKI, I/R induced apparent renal injury at 24 hr, presenting significant increase of Cr and BUN in our research. With the condition of IR, hyperlipidemia could increase Cr and BUN, they were decreased by pre-treatment with TIIA (Figure 1A). In comparison, I/R induced apparent renal injury at 48 hr in previous studies [22].

Although there are various types of researches about IR-induced AKI, the potential mechanism of injury has not been absolutely understood [38]. Renal I/R damage evokes correlated series of events in the kidney, which lead to renal damage and even death of renal tubular cells. Tubular cell damage and endothelial dysfunction via the apoptotic pathway, imbalance between oxidation and antioxidation, ATP depletion, and proinflammatory cytokines have been explored [39]. Mitochondrial apoptotic signaling pathways (NF-jB/p53/PUMA) perform an important function in renal I/R injury, while renal I/R can induce mitochondrial dysfunction [40]. In this study, IR could induce the abnormal mitochondrial morphology (membrane rupture or swelling), and then increase the percentage of damaged mitochondria; whereas TIIA could protect the mitochondria from renal IR combined with hyperlipidemia (Figure 4C). In the future, we will study the correlation between NF-jB/p53/PUMA and PI3K/Akt/Bad.

Mitochondrial oxygen reduction equilibrium and homeostasis play a crucial role in the ischemic AKI of pathophysiological changes. Impaired mitochondrial function and imbalance between oxidation and antioxidation are the key factors of renal I/R injury [41]. With the ischemic condition, anoxia and substrates repress mitochondrial respiration. Then, renal tissues must change to glycolysis metabolism, which dramatically decreases the ability for producing ATP quickly. Cell swelling caused by ATP deficiency raises an osmotic gradient, which actuates water into the mitochondrial matrix [42]. Furthermore, hyperlipidemia induces a marked generation of deleterious mitochondrial ROS cand causes oxidative damage, which activates caspase-dependent apoptosis through mitochondrial pathway; this further impairs MMP and then raises renal podocyte apoptosis [43]. The outcomes of mitochondrial dysfunction induces renal IR injury. In our research, we built model of renal IRI in obese rats followed by pre-treatment with TIIA. We detected the MMP, opening of the mPTP (%), mtDNA, ROS, RCR, ATP, and so on. Those indexes were thought to be the sign of evaluating mitochondrial function. In our study, RCR, MMP, oxygen consumption rate, ATP, and mitochondrial respiratory chain complex enzymes were reduced by IR/IR (obese). However, ROS and opening of the mPTP (%) were increased by IR/IR (obese). In comparison, pre-treatment with TIIA could improve the above indexes of mitochondrial function (Figure 4A, 4B).

The copy number of mtDNA of every mitochondrion remains steady. Therefore, total copy number of mtDNA can be used to appraise mitochondrial quantity [44, 45]. Despite the fact that the mechanism of scathed mtDNA is ambiguous, mtDNA is close to respiratory chain. Therefore, mtDNA is more susceptible when revealed to oxidative reaction. We studied the damage degree of mtDNA via counting the ratio of long/short fragments in this study. The ratio of long/short fragments was obviously increased by IR (obese); but after pre-treatment with TIIA, the ratio could be increased (Figure 4A).

As a significant adaptation of exposing to chronic energy deficiency, mitochondrial biogenesis could be operated by many complicated factors, such as Tfam and Nrf1. The Nrf1 can foster the expression of transcription of nuclei-encoded mitochondrial proteins. Containing these refers to oxidative phosphorylation and respiratory complexes. Tfam increases gene transcription and DNA replication in mitochondria through directly binding to the mitochondrial genome. As a crucial transcriptional co-activator, PGC-1α can operate critical elements containing Tfam and Nrf1, which can increase mitochondrial biogenesis [46]. When the levels of above gene vary, mitochondrial biogenesis will get chaotic. As shown in Figure 5, IR could reduce the expression of Nrf1, Tfam, and PGC-1α. TIIA increased the level of Nrf1, PGC-1α, and Tfam. After TIIA interference, an ample power supply reduced ROS and increased mitochondria biogenesis elements, which performed synergistically to give rise to improved lack of intracellular energy furnish. Normally, deleterious irritation containing aging, energy limitation, and oxidizing reaction can damage mitochondria to be closed up in autophagosomes, fused to lysosomes, and finally degraded. Abnormalities of lautophagy in mitochondria can raise the number of damaged mitochondria [25].

Typically, mitochondria go through the dynamic course containing fission and fusion. The course is significant to preserve stable varieties of dimensions, fashion, and networks in mitochondria, which are governed by related proteins containing Drp1 and Mfn1/2 [47]. Our results (Figure 5) showed that the expressions of Drp1 and Mfn1/2 changed in opposite orientations following IR, demonstrating a disordered balance of fission-fusion of mitochondria. Mfn1/2 and Drp1 were thought to perform a critical function respectively on mitochondrial fusion and fission. As such, we found an increase of Mfn1/2 and a decrease of Drp1 following renal IR in renal tissues.

PI3K/Akt/Bad signalling pathway plays a crucial function in repressing apoptosis regulated by mitochondria [17]. Because of their close connection, the effect of PI3K was studied in this study. As a phosphatidylinositol kinase, PI3K can activate phosphatidylinositol kinase and threonine-specific proteins kinase [48]. After being activated, phosphatidylinositol family members in cell membrane can be phosphorylated and Akt (downstream molecule) can be activated, which can then activate Akt phosphorylates (Ser136/Ser112) residues Bad protein [49]. Phosphorylated Bad segregates from the apoptosis-promoting complex and then shapes the protein complex (14-3-3), inducing inactivating the apoptosis-promoting role and then restraining apoptosis [50]. The results showed that TIIA effectively upregulated the PI3K/Akt/Bad pathway proteins and then upregulates PI3K and p-Akt proteins and downregulates the proteins (Cyt-c and PARP) (Figure 6). The above results showed TIIA could adjust mitochondrial function, and then inhibit cells apoptosis caused by renal IRI through activating PI3K/Akt/Bad pathway. Immunofluorescence results of the proteins (Bax/Bcl-2) expression of kidney tissues showed that renal IR increased Bax and decreased Bcl-2. Nevertheless, Bax can be decreased, while Bcl-2 increased, by pre-treatment with TIIA (Figure 3).

**Figure 7:**
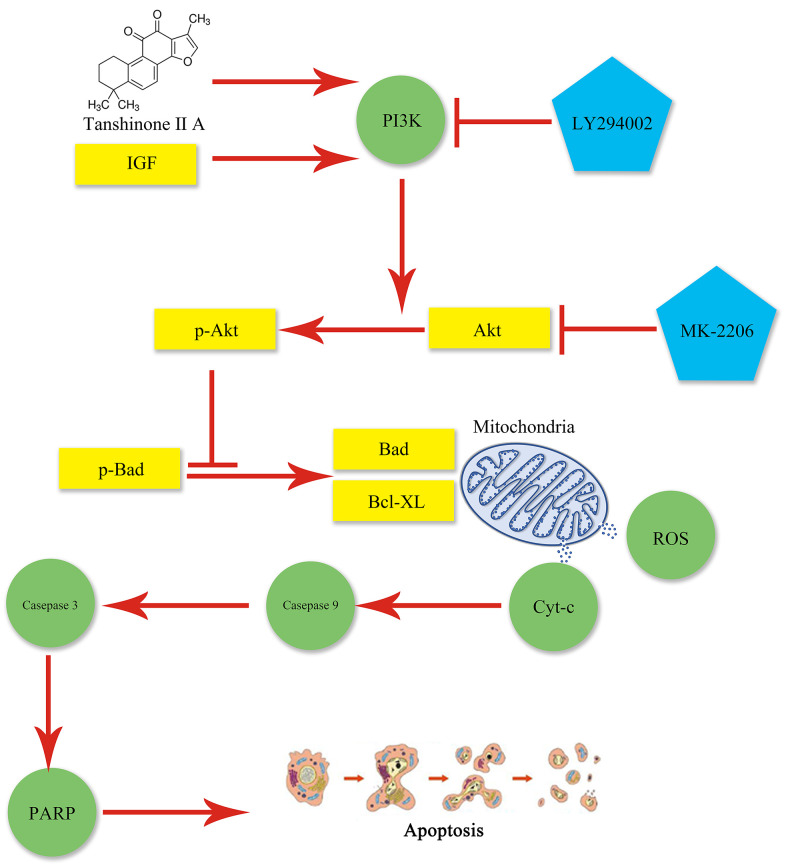
**Graphical abstract.** Mitochondrial dysfunction occurs following AKI and may lead to apoptosis, which can be aggravated by obesity. TIIA can relieve apoptosis by improving mitochondrial function via activating PI3K/Akt/Bad pathway in obesity rats.

In our study, we used isolated mitochondria and showed that IR accelerated ROS, which injured mtDNA, and by such means injured mitochondrial respiratory function, biogenesis, and dynamic function, and produced ROS. The abnormal mitochondria were caused by opening mPTP following renal IR. The opening of mPTP could induce flow back of proton from mitochondrial membrane space to matrix, hence decreasing MMP and ATP and leading to metabolic abnormalities. The reducing of ATP and MMP and the increasing of Cyt-c were caused by opening mPTP, inducing metabolic abnormalities and apoptosis. Nevertheless, pre-treatment with TIIA could restrain apoptosis via regulating mitochondrial function through activating the PI3K/Akt/Bad pathway of obese rats (Figure 7).

## References

[1] Koyner JL, Cerdá J, Goldstein SL, Jaber BL, Liu KD, Shea JA, Faubel S, and Acute Kidney Injury Advisory Group of the American Society of Nephrology. The daily burden of acute kidney injury: a survey of U.S. nephrologists on World Kidney Day. Am J Kidney Dis. 2014; 64:394–401. 10.1053/j.ajkd.2014.03.018 24815216

[2] Ploeg RJ, van Bockel JH, Langendijk PT, Groenewegen M, van der Woude FJ, Persijn GG, Thorogood J, Hermans J. Effect of preservation solution on results of cadaveric kidney transplantation. The European Multicentre Study Group. Lancet. 1992; 340:129–37. 10.1016/0140-6736(92)93212-6 1352564

[3] Hafner S, Hillenbrand A, Knippschild U, Radermacher P. The obesity paradox and acute kidney injury: beneficial effects of hyper-inflammation? Crit Care. 2013; 17:1023. 10.1186/cc13152 24326122 PMC4059416

[4] Kelz RR, Reinke CE, Zubizarreta JR, Wang M, Saynisch P, Even-Shoshan O, Reese PP, Fleisher LA, Silber JH. Acute kidney injury, renal function, and the elderly obese surgical patient: a matched case-control study. Ann Surg. 2013; 258:359–63. 10.1097/SLA.0b013e31829654f3 23676533 PMC3931547

[5] Wilson PW, D’Agostino RB, Levy D, Belanger AM, Silbershatz H, Kannel WB. Prediction of coronary heart disease using risk factor categories. Circulation. 1998; 97:1837–47. 10.1161/01.cir.97.18.1837 9603539

[6] Dalrymple LS, Kaysen GA. The effect of lipoproteins on the development and progression of renal disease. Am J Nephrol. 2008; 28:723–31. 10.1159/000127980 18434711

[7] Kwan BC, Kronenberg F, Beddhu S, Cheung AK. Lipoprotein metabolism and lipid management in chronic kidney disease. J Am Soc Nephrol. 2007; 18:1246–61. 10.1681/ASN.2006091006 17360943

[8] Nitzan M. Abnormalities of carbohydrate and lipid metabolism in experimentally induced acute uremia. Nutr Metab. 1973; 15:187–91. 10.1159/000175439 4352215

[9] Kwong JQ, Molkentin JD. Physiological and pathological roles of the mitochondrial permeability transition pore in the heart. Cell Metab. 2015; 21:206–14. 10.1016/j.cmet.2014.12.001 25651175 PMC4616258

[10] Sui S, Tian J, Gauba E, Wang Q, Guo L, Du H. Cyclophilin D regulates neuronal activity-induced filopodiagenesis by fine-tuning dendritic mitochondrial calcium dynamics. J Neurochem. 2018; 146:403–15. 10.1111/jnc.14484 29900530 PMC6107423

[11] Yin X, Yin Y, Cao FL, Chen YF, Peng Y, Hou WG, Sun SK, Luo ZJ. Tanshinone IIA attenuates the inflammatory response and apoptosis after traumatic injury of the spinal cord in adult rats. PLoS One. 2012; 7:e38381. 10.1371/journal.pone.0038381 22675554 PMC3365897

[12] Xu W, Yang J, Wu LM. Cardioprotective effects of tanshinone IIA on myocardial ischemia injury in rats. Pharmazie. 2009; 64:332–6. 19530445

[13] Zhang SZ, Ye ZG, Xia Q, Zhang W, Bruce I. Inhibition of Mitochondrial Permeability Transition Pore: A Possible Mechanism for Cardioprotection Conferred by Pretreatment with Tanshinone IIA. Conf Proc IEEE Eng Med Biol Soc. 2005; 2005:2276–9. 10.1109/IEMBS.2005.1616918 17282687

[14] Zhu B, Zhai Q, Yu B. Tanshinone IIA protects rat primary hepatocytes against carbon tetrachloride toxicity via inhibiting mitochondria permeability transition. Pharm Biol. 2010; 48:484–7. 10.3109/13880200903179699 20645787

[15] Xu YM, Ding GH, Huang J, Xiong Y. Tanshinone IIA pretreatment attenuates ischemia/reperfusion-induced renal injury. Exp Ther Med. 2016; 12:2741–6. 10.3892/etm.2016.3674 27698779 PMC5038172

[16] Rodgers SJ, Ferguson DT, Mitchell CA, Ooms LM. Regulation of PI3K effector signalling in cancer by the phosphoinositide phosphatases. Biosci Rep. 2017; 37:BSR20160432. 10.1042/BSR20160432 28082369 PMC5301276

[17] Zeng KW, Wang XM, Ko H, Kwon HC, Cha JW, Yang HO. Hyperoside protects primary rat cortical neurons from neurotoxicity induced by amyloid β-protein via the PI3K/Akt/Bad/Bcl(XL)-regulated mitochondrial apoptotic pathway. Eur J Pharmacol. 2011; 672:45–55. 10.1016/j.ejphar.2011.09.177 21978835

[18] Neto JS, Nakao A, Kimizuka K, Romanosky AJ, Stolz DB, Uchiyama T, Nalesnik MA, Otterbein LE, Murase N. Protection of transplant-induced renal ischemia-reperfusion injury with carbon monoxide. Am J Physiol Renal Physiol. 2004; 287:F979–89. 10.1152/ajprenal.00158.2004 15292046

[19] Alzoubi KH, Abdul-Razzak KK, Khabour OF, Al-Tuweiq GM, Alzubi MA, Alkadhi KA. Adverse effect of combination of chronic psychosocial stress and high fat diet on hippocampus-dependent memory in rats. Behav Brain Res. 2009; 204:117–23. 10.1016/j.bbr.2009.05.025 19482049

[20] Tawfik MK. Renoprotective activity of telmisartan versus pioglitazone on ischemia/reperfusion induced renal damage in diabetic rats. Eur Rev Med Pharmacol Sci. 2012; 16:600–9. 22774400

[21] Ali SI, Alhusseini NF, Atteia HH, Idris RA, Hasan RA. Renoprotective effect of a combination of garlic and telmisartan against ischemia/reperfusion-induced kidney injury in obese rats. Free Radic Res. 2016; 50:966–86. 10.1080/10715762.2016.1211644 27405440

[22] Changizi-Ashtiyani S, Hafazeh L, Ghasemi F, Najafi H, Babaei S, JalallyMashayekhi F, Hoseini SJ, Bastani B. The effect of adipose-derived mesenchymal stem cells on renal function and histopathology in a rat model of ischemia-reperfusion induced acute kidney injury. Iran J Basic Med Sci. 2020; 23:999–1006. 10.22038/ijbms.2020.40334.9601 32952945 PMC7478256

[23] Tai H, Tong YJ, Yu R, Yu Y, Yao SC, Li LB, Liu Y, Cui XZ, Kuang JS, Meng XS, Jiang XL. A possible new activator of PI3K-Huayu Qutan Recipe alleviates mitochondrial apoptosis in obesity rats with acute myocardial infarction. J Cell Mol Med. 2022; 26:3423–45. 10.1111/jcmm.17353 35567290 PMC9189350

[24] Zhou J, Ma X, Shi M, Chen C, Sun Y, Li J, Xiong Y, Chen J, Li F. Serum metabolomics analysis reveals that obvious cardioprotective effects of low dose Sini decoction against isoproterenol-induced myocardial injury in rats. Phytomedicine. 2017; 31:18–31. 10.1016/j.phymed.2017.01.009 28606513

[25] Luan G, Li G, Ma X, Jin Y, Hu N, Li J, Wang Z, Wang H. Dexamethasone-Induced Mitochondrial Dysfunction and Insulin Resistance-Study in 3T3-L1 Adipocytes and Mitochondria Isolated from Mouse Liver. Molecules. 2019; 24:1982. 10.3390/molecules24101982 31126054 PMC6572075

[26] Li X, Jia P, Huang Z, Liu S, Miao J, Guo Y, Wu N, Jia D. Lycopene protects against myocardial ischemia-reperfusion injury by inhibiting mitochondrial permeability transition pore opening. Drug Des Devel Ther. 2019; 13:2331–42. 10.2147/DDDT.S194753 31371925 PMC6635826

[27] Cioffi F, Senese R, Lasala P, Ziello A, Mazzoli A, Crescenzo R, Liverini G, Lanni A, Goglia F, Iossa S. Fructose-Rich Diet Affects Mitochondrial DNA Damage and Repair in Rats. Nutrients. 2017; 9:323. 10.3390/nu9040323 28338610 PMC5409662

[28] Gilmer LK, Ansari MA, Roberts KN, Scheff SW. Age-related mitochondrial changes after traumatic brain injury. J Neurotrauma. 2010; 27:939–50. 10.1089/neu.2009.1181 20175672 PMC2943941

[29] Wagner EM. Monitoring gene expression: quantitative real-time rt-PCR. Methods Mol Biol. 2013; 1027:19–45. 10.1007/978-1-60327-369-5_2 23912981

[30] Livak KJ, Schmittgen TD. Analysis of relative gene expression data using real-time quantitative PCR and the 2(-Delta Delta C(T)) Method. Methods. 2001; 25:402–8. 10.1006/meth.2001.1262 11846609

[31] Agarwal KC. Therapeutic actions of garlic constituents. Med Res Rev. 1996; 16:111–24. 10.1002/(SICI)1098-1128(199601)16:1<111::AID-MED4>3.0.CO;2-5 8788216

[32] Song M, Hang TJ, Zhang ZX. [Pharmacokinetic interactions between the main components in the extracts of Salvia miltiorrhiza Bge. in rat]. Yao Xue Xue Bao. 2007; 42:301–7. 17520831

[33] Zhang Z, He H, Qiao Y, Huang J, Wu Z, Xu P, Yin D, He M. Tanshinone IIA Pretreatment Protects H9c2 Cells against Anoxia/Reoxygenation Injury: Involvement of the Translocation of Bcl-2 to Mitochondria Mediated by 14-3-3η. Oxid Med Cell Longev. 2018; 2018:3583921. 10.1155/2018/3583921 30050654 PMC6046124

[34] Lemoine S, Pillot B, Augeul L, Rabeyrin M, Varennes A, Normand G, Baetz D, Ovize M, Juillard L. Dose and timing of injections for effective cyclosporine A pretreatment before renal ischemia reperfusion in mice. PLoS One. 2017; 12:e0182358. 10.1371/journal.pone.0182358 28796779 PMC5552114

[35] Lemoine S, Pillot B, Rognant N, Augeul L, Rayberin M, Varennes A, Laville M, Ovize M, Juillard L. Postconditioning with cyclosporine a reduces early renal dysfunction by inhibiting mitochondrial permeability transition. Transplantation. 2015; 99:717–23. 10.1097/TP.0000000000000530 25793558

[36] Oh J, Rabb H. Adiponectin: an enlarging role in acute kidney injury. Kidney Int. 2013; 83:546–8. 10.1038/ki.2012.479 23538694

[37] Wahba IM, Mak RH. Obesity and obesity-initiated metabolic syndrome: mechanistic links to chronic kidney disease. Clin J Am Soc Nephrol. 2007; 2:550–62. 10.2215/CJN.04071206 17699463

[38] Han SJ, Lee HT. Mechanisms and therapeutic targets of ischemic acute kidney injury. Kidney Res Clin Pract. 2019; 38:427–40. 10.23876/j.krcp.19.062 31537053 PMC6913588

[39] Wu D, Chen X, Ding R, Qiao X, Shi S, Xie Y, Hong Q, Feng Z. Ischemia/reperfusion induce renal tubule apoptosis by inositol 1,4,5-trisphosphate receptor and L-type Ca2+ channel opening. Am J Nephrol. 2008; 28:487–99. 10.1159/000113107 18185015

[40] Chen C, Yao W, Wu S, Zhou S, Ge M, Gu Y, Li X, Chen G, Bellanti JA, Zheng SG, Yuan D, Hei Z. Crosstalk Between Connexin32 and Mitochondrial Apoptotic Signaling Pathway Plays a Pivotal Role in Renal Ischemia Reperfusion-Induced Acute Kidney Injury. Antioxid Redox Signal. 2019; 30:1521–38. 10.1089/ars.2017.7375 29790387 PMC7364332

[41] Rojas-Morales P, León-Contreras JC, Aparicio-Trejo OE, Reyes-Ocampo JG, Medina-Campos ON, Jiménez-Osorio AS, González-Reyes S, Marquina-Castillo B, Hernández-Pando R, Barrera-Oviedo D, Sánchez-Lozada LG, Pedraza-Chaverri J, Tapia E. Fasting reduces oxidative stress, mitochondrial dysfunction and fibrosis induced by renal ischemia-reperfusion injury. Free Radic Biol Med. 2019; 135:60–7. 10.1016/j.freeradbiomed.2019.02.018 30818054

[42] Kaasik A, Safiulina D, Zharkovsky A, Veksler V. Regulation of mitochondrial matrix volume. Am J Physiol Cell Physiol. 2007; 292:C157–63. 10.1152/ajpcell.00272.2006 16870828

[43] Liu T, Chen XM, Sun JY, Jiang XS, Wu Y, Yang S, Huang HZ, Ruan XZ, Du XG. Palmitic Acid-Induced Podocyte Apoptosis via the Reactive Oxygen Species-Dependent Mitochondrial Pathway. Kidney Blood Press Res. 2018; 43:206–19. 10.1159/000487673 29490300

[44] Yan C, Duanmu X, Zeng L, Liu B, Song Z. Mitochondrial DNA: Distribution, Mutations, and Elimination. Cells. 2019; 8:379. 10.3390/cells8040379 31027297 PMC6523345

[45] Clay Montier LL, Deng JJ, Bai Y. Number matters: control of mammalian mitochondrial DNA copy number. J Genet Genomics. 2009; 36:125–31. 10.1016/S1673-8527(08)60099-5 19302968 PMC4706993

[46] Kelly DP, Scarpulla RC. Transcriptional regulatory circuits controlling mitochondrial biogenesis and function. Genes Dev. 2004; 18:357–68. 10.1101/gad.1177604 15004004

[47] de Brito OM, Scorrano L. Mitofusin 2 tethers endoplasmic reticulum to mitochondria. Nature. 2008; 456:605–10. 10.1038/nature07534 19052620

[48] Wang Y, Yuan Y, Gao Y, Li X, Tian F, Liu F, Du R, Li P, Wang F, Xu S, Wu X, Wang C. MicroRNA-31 regulating apoptosis by mediating the phosphatidylinositol-3 kinase/protein kinase B signaling pathway in treatment of spinal cord injury. Brain Dev. 2019; 41:649–61. 10.1016/j.braindev.2019.04.010 31036380

[49] Fang X, Yu S, Eder A, Mao M, Bast RC Jr, Boyd D, Mills GB. Regulation of BAD phosphorylation at serine 112 by the Ras-mitogen-activated protein kinase pathway. Oncogene. 1999; 18:6635–40. 10.1038/sj.onc.1203076 10597268

[50] Sakamaki J, Daitoku H, Ueno K, Hagiwara A, Yamagata K, Fukamizu A. Arginine methylation of BCL-2 antagonist of cell death (BAD) counteracts its phosphorylation and inactivation by Akt. Proc Natl Acad Sci USA. 2011; 108:6085–90. 10.1073/pnas.1015328108 21444773 PMC3076815

[51] Percie du Sert N, Hurst V, Ahluwalia A, Alam S, Avey MT, Baker M, Browne WJ, Clark A, Cuthill IC, Dirnagl U, Emerson M, Garner P, Holgate ST, et al. The ARRIVE guidelines 2.0: Updated guidelines for reporting animal research. Exp Physiol. 2020; 105:1459–66. 10.1113/EP088870 32666546 PMC7610926

